# Supplementary material for: Integrated single-cell multiomic profiling of caudate nucleus suggests key mechanisms in alcohol use disorder
Source: Nat Commun. 2025 Oct 13;16:9070. doi: 10.1038/s41467-025-64136-0 (PMC12518533; doi:10.1038/s41467-025-64136-0)
Supplement: Supplementary file 1 — Supplementary Information [file 41467_2025_64136_MOESM1_ESM.pdf]

Supplementary Information

Gene Expression Correlation Between Cell Types

The differences in gene expression within D1 neurons and D2 neurons were highly correlated with each other (Pearson correlation = 0.83), while differences within other neuronal and nonneuronal cell types were more weakly correlated (Pearson correlation ranges = 0.10-0.46 for neuronal cell types and 0.05-0.27 for non-neuronal cell types; Supplementary Fig. 5b, Supplementary Data 8). Interestingly, expression differences within D1/D2 neurons were much less correlated with either D1 or D2 neurons (r = 0.41 and 0.39 respectively).

Chromatin Accessibility Peaks and Correlation

Thirty-six percent of all open chromatin regions were shared among neuronal and non-neuronal cell types, while 34% of the peaks were unique to neurons and 30% unique to non-neurons (Supplementary Fig. 6c).

For comparing similarity of peaks called between cell types, the Jaccard index was used, defined here as the number of peaks in one cell type overlapping a peak in the other cell type, divided by the union of the peaks in both cell types. D1 neurons and D2 neurons had very similar open chromatin regions (Jaccard index = 0.8) and were less similar to D1/D2 neurons (Jaccard index = 0.47). Astrocytes, oligodendrocytes, and OPCs had moderately similar open chromatin regions, with Jaccard indices of approximately 0.4 between these cell types (Supplementary Fig. 6d, Supplementary Data 10).

Enrichment of DEGs with alcohol-related GWAS Genes

Several large-scale GWAS have found genetic loci associated with AUD-related traits: 496 independent loci associated with number of drinks per week,4 and 90 independent loci associated with PAU,6 including 5 loci associated with both traits. There are 3,406 and 668 genes, respectively, within these loci, of which 448 were associated with both traits, a total of 3,626 unique genes. Of these, 189 were differentially expressed (FDR < 0.05) in astrocytes in our snRNA-seq data, 395 in oligodendrocytes, 90 in D1 neurons, and 81 in D2 neurons.

Gene set enrichment analysis showed that differentially expressed genes were enriched for GWAS-implicated genes in all GABAergic neuronal cell types, as well as ependymal cells. Namely D1 MSNs, D2 MSNs, FS, LTS, and CCK interneurons, and ependymal cells exhibited positive enrichment scores for drinks per week-associated genes, suggesting that genes associated with increased alcohol consumption are upregulated in these cell types in AUD. (FDR < 0.05). CR interneurons had negative enrichment scores for drinks per week genes, suggesting downregulation of these genes in individuals with AUD (FDR < 0.05). Additionally, D1/D2 MSNs had significantly negative enrichment for PAU genes in AUD, also suggesting downregulation of these genes in individuals with AUD (FDR = 0.034). Therefore. in these cell

types, it is plausible that AUD genetics directly influences differentially expressed genes that could drive the phenotype.

The remaining cell types were not highly significantly enriched for either of the GWAS gene sets in AUD (FDR > 0.05).

Supplementary Gene Regulatory Network Analysis

Gene Regulatory Module Formation

To detect key TF-TG subnetworks (modules) from the cell population TF–TG trans-regulation, we used non-negative matrix factorization (NMF). Before matrix factorization, We normalized the trans-regulatory potential matrix by standardizing each row (TF) and each column (TG) independently. The standardization of each TF ensures that for each TF, the average regulatory potential across TGs becomes zero, and the variation in regulatory potential across genes has a standard deviation of one. The same normalization was applied to each TG, so that the effect of the regulator side was also normalized. We took the average of these two standardized matrices and set the negative values to zeros, which was used for downstream analysis. Next, we performed NMF on the preprocessed matrix to decompose it into two non-negative matrices, WW and HH, representing module membership of TGs and TFs, respectively. WW is a mm by kk module weight matrix for TF, representing module weight of TF, where m is the number of TF. HH is a kk by nn matrix, representing module weight for TGs, where n is the number of TG. To assign TF and TG into specific modules, we normalized the module weight matrix to equal sum for different modules. For each gene, we converted the normalized weight matrix into proportions by dividing the sum of weights across modules. We sorted genes based on their highest proportion across all modules to select the top 10% of genes and assign them to modules for which the gene has the largest score.

This procedure was applied to TF module W matrix and the TG module HH matrix. Here, we identified 10 trans-regulatory modules.

To uncover AUD-associated regulatory programs in each cell type, we performed differential module expression analysis. We first pre-processed the pseudo-bulk gene expression count matrix by: (1) normalizing for cell depth, (2) log-transforming, and (3) z-scoring expression across all donors. We then estimated module activity in each donor as the mean expression of module genes. Using a two-tailed two-sample t-test, we identified differentially active modules between individuals with and without AUD.

Gene Regulatory Module Results

Among the 10 modules detected, several showed differential expression between those with and without AUD (Supplementary Fig. 7a, Supplementary Data 14): module 2 genes in astrocytes, module 7 in D1/D2 MSNs (also marginally significant in oligodendrocytes, FDR = 0.054), and module 1 in microglia (FDR < 0.05). Module 7 target genes were enriched for several pathways from GP Cellular Component database, including collagen-containing

extracellular matrix (FDR = 0.003), vesicle (FDR = 0.007) and basement membrane (FDR = 0.007).

Notably, genes in module 2 in astrocytes and in module 1 in microglia both had lower expression in individuals with AUD and were enriched for genes from the PAU GWAS. Module 1 contained only one transcription factor, ZBTB16, which is a negative regulator of inflammation, including in microglia. Module 1 contained 6 target genes that were enriched for neurodegeneration in the Human Phenotype Ontology (HP:0002180, FDR = 0.017), including KLB, a gene associated with alcohol consumption.

Module 2, with lower expression in astrocytes from those with AUD, contained 24 transcription factors and 451 target genes. To determine which of these genes were more likely to be regulated together, we performed weighted gene co-expression network analysis (WGCNA). 55 of astrocyte single-nucleus data, which revealed ten groups of co-expressed genes (Supplementary Data 15). Co-expression group 3 (Co.E3) was enriched for differentially expressed genes, particularly those with lower expression in individuals with AUD. There were 75 genes in Co.E3 that overlapped with regulatory module 2 (Supplementary Fig. 7b), suggesting that these genes not only show similar expression patterns but also have similar patterns of regulation. Functional enrichment analysis identified several enriched pathways, including pathways relating to glutamatergic synapses, nervous system development, and negative regulation of Wnt signaling pathways from GO Biological Process and Cellular Component (Supplementary Fig. 7c).

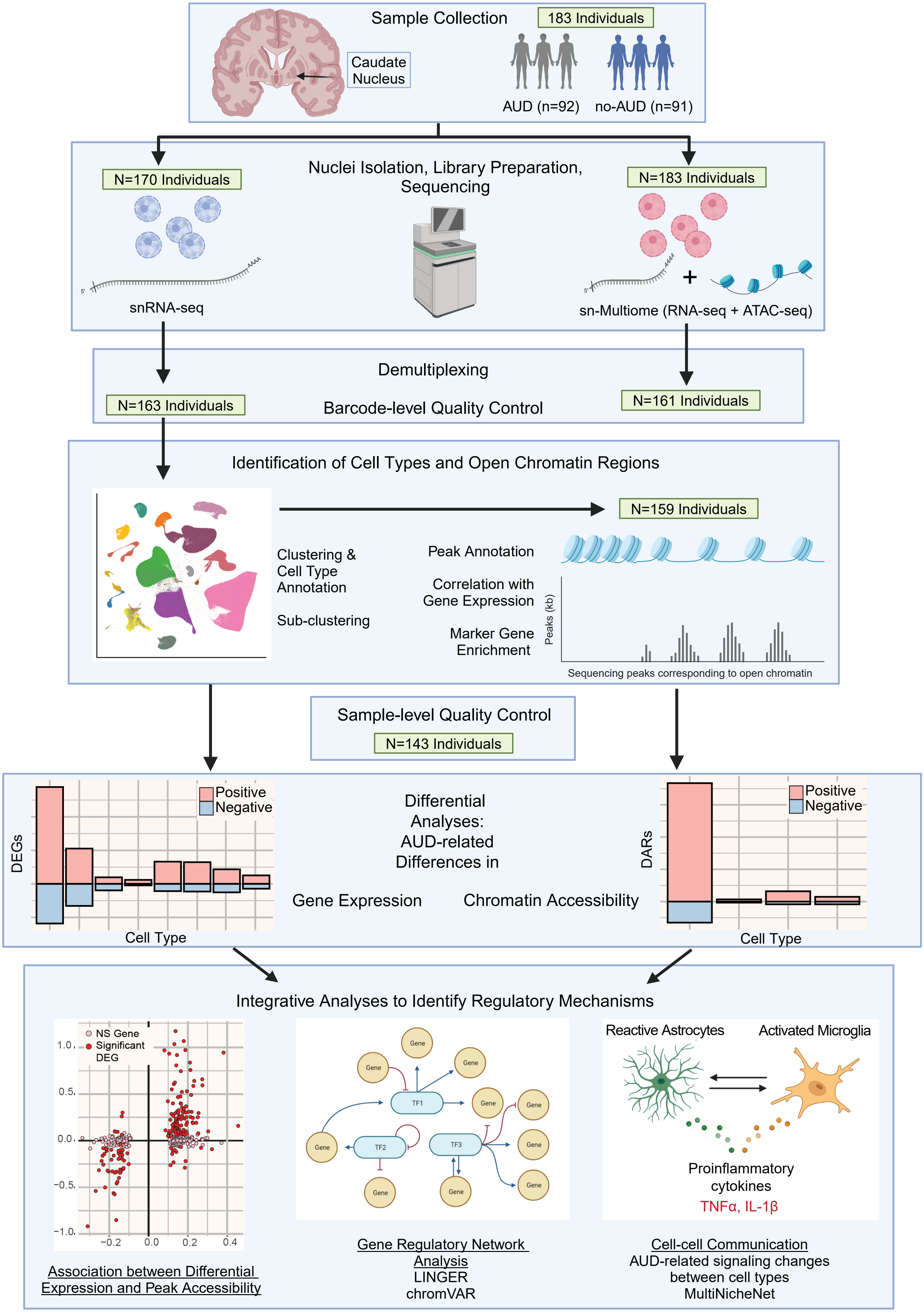

Supplementary Figure 1**:** Quality Control and Analysis Overview. Blue boxes denote broad steps of analysis pipeline, and green boxes denote number of individuals retained after various quality control steps. See Methods for details. Created in BioRender. Green, N. (2026) https://BioRender.com/1ijh8jd

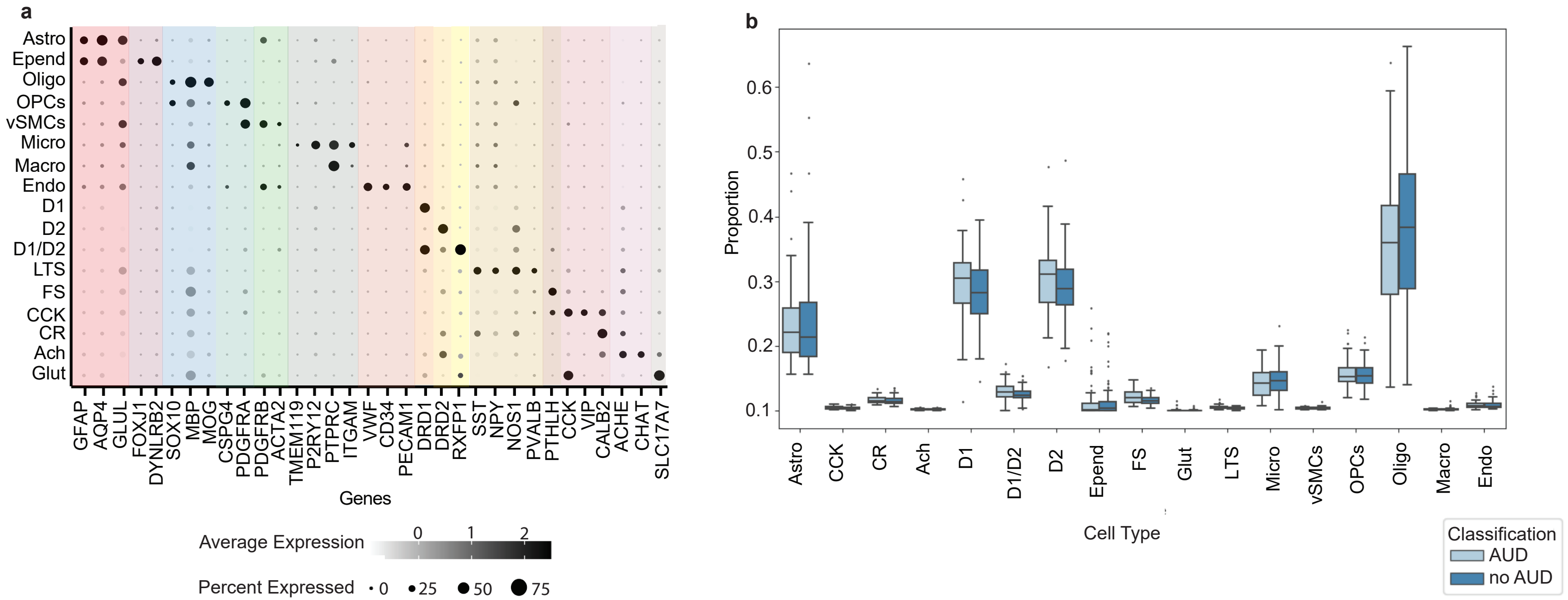

Supplementary Figure 2: **a** Normalized expression in each cell type of the marker genes used to identify cell types. Dot size corresponds to the percentage of cells expressing the gene; dot intensity indicates average gene expression level. 'Average Expression' denotes the mean log-normalized expression level across cells in each subcluster, scaled for each gene, and 'Percent Expressed' denotes the percentage of cells in which the log-normalized expression of the gene is greater than zero. b Proportion of each cell type for each of the 163 individuals used for clustering and cell type annotation, grouped by AUD classification. Center of boxes denote median log Enrichment Score, with box boundaries denoting Q1 (25th percentile) and Q3 (75th Percentile). Bottom whisker edge denotes Q1 – 1.5*Interquartile Range (IQR), and top whisker denotes Q3 + 1.5*IQR. Here and following, "log" denotes natural logarithm. Source data are provided as a Source Data file. Cell type abbreviations:

Astro: Astrocytes
CCK: CCK+ interneurons
CR: CR+ interneurons
Ach: Cholinergic neurons
D1: D1 Medium spiny neurons
D2: D2 Medium spiny neurons
D1/D2: D1/D2 hybrid medium spiny neurons
Epend: Ependymal cells
FS: Fast Spiking (PV+) interneurons
LTS: Low-threshold spiking (SST+) interneurons
Micro: Microglia
vSMCs: Vascular smooth muscle cells
OPCs: Oligodendrocyte progenitor cells
Oligo: Oligdendrocytes
Macro: Macrophages
Endo: Endothelial Cells

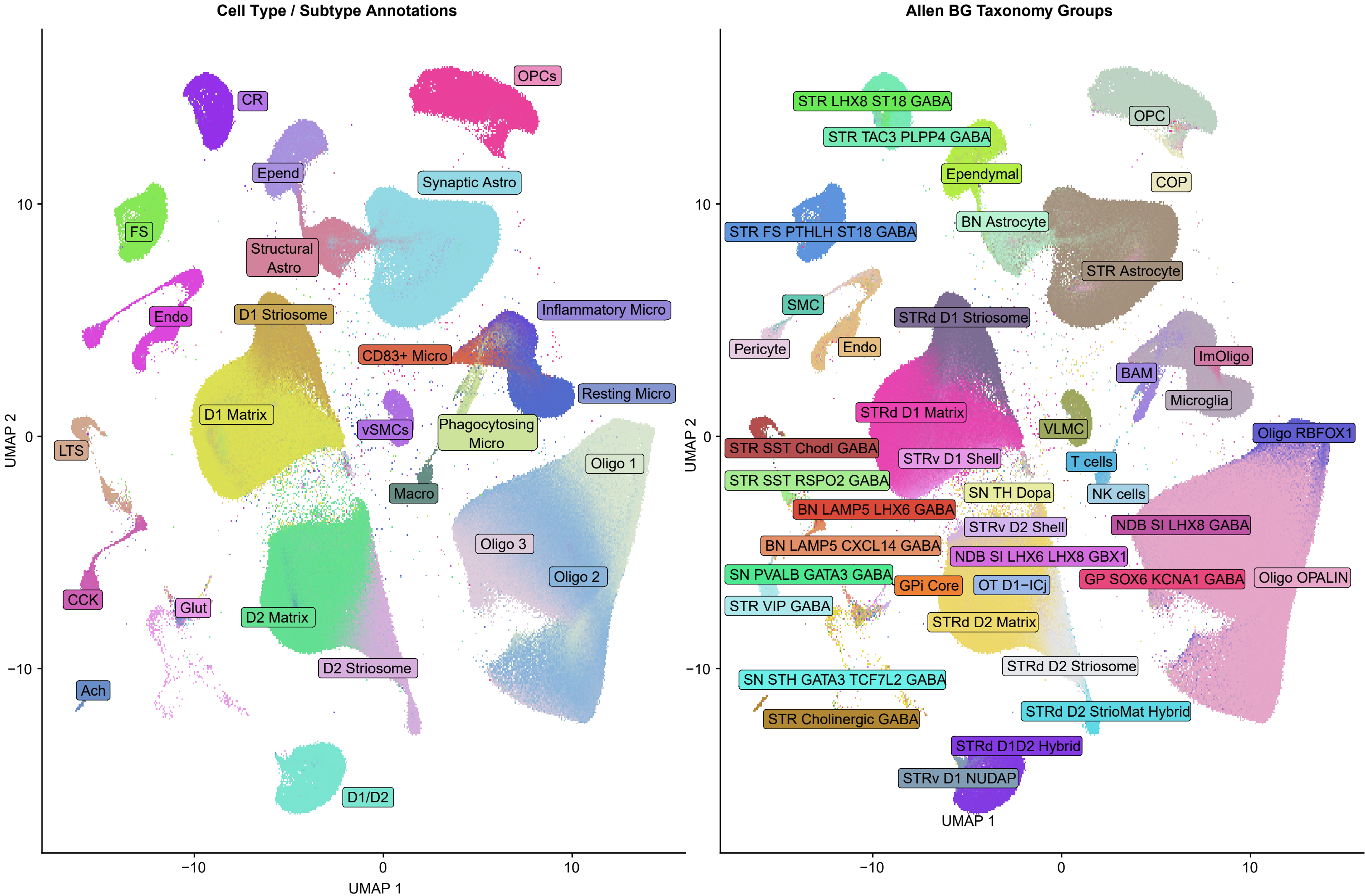

Supplementary Figure 3: Cell type annotations compared to Allen Basal Ganglia Consensus Cell Type Atlas. UMAP of 1,121,762 nuclei used for differential analyses, visualization shown is based on the snRNA-seq profile. Left: Nuclei are labeled by cell type as assigned in “Clustering reveals 17 well-established and well-differentiated striatal cell types”. Cell types: cholinergic neurons (Ach), astrocytes (Astro), cholecystokinin-expressing interneurons (CCK), calretinin-expressing interneurons (CR), D1-type medium spiny neurons (D1), D2-type medium spiny neuron (D2), medium spiny neurons expressing both D1 and D2 receptors (D1/D2), endothelial cells (Endo), ependymal cells (Epend), fast-spiking interneurons (FS), glutamatergic neurons (Glut), low-threshold spiking interneurons (LTS), non-microglial macrophages (Macro), microglia (Micro), oligodendrocytes (Oligo), oligodendrocyte progenitor cells (OPCs), and vascular smooth muscle cells (vSMCs). Right: Nuclei are labeled as mapped on the Allen Basal Ganglia (BG) Consensus Taxonomy using Allen Institute’s Cell Type Mapper. Labels shown are the “Group” label in the BG taxonomy. Source data are provided as a Source Data file.

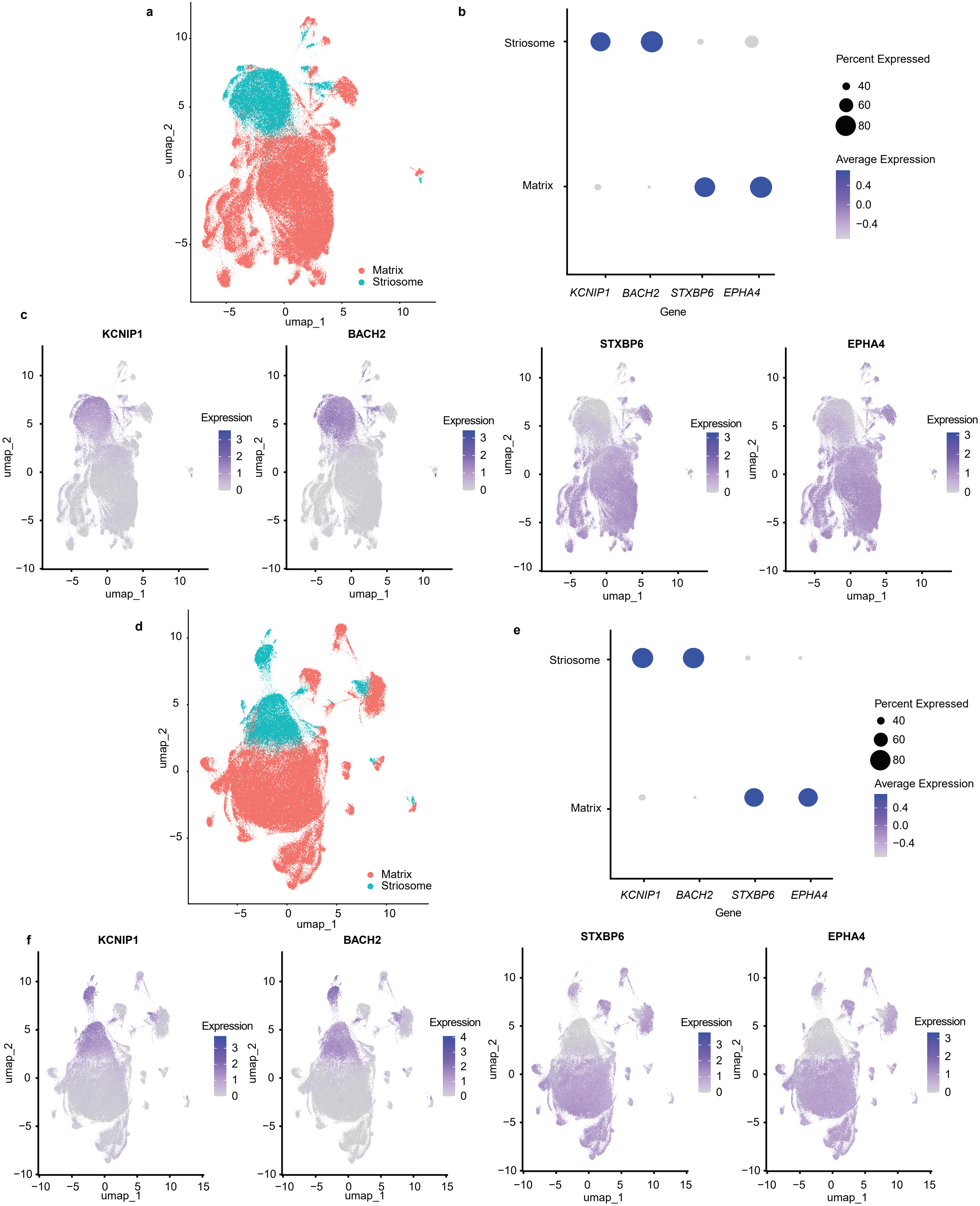

Supplementary Figure 4: D1 and D2 Medium Spiny Neurons Subtypes. **a**, UMAP of 200,729 D1 medium spiny neurons, colored by compartment (either matrix or striosome). **b** Dot pot of expression and prevalence of representative marker genes for matrix and striosome compartments. ‘Average Expression’ denotes the mean log-normalized expression level across cells in each subcluster, scaled for each gene, and ‘Percent Expressed’ denotes the percentage of cells in which the log-normalized expression of the gene is greater than zero. **c** UMAP of D1 cells, colored by log-normalized expression of marker genes used to assign compartment. **d-f** as a-c, for 209,742 D2 medium spiny neurons. Source data are provided as a Source Data file.

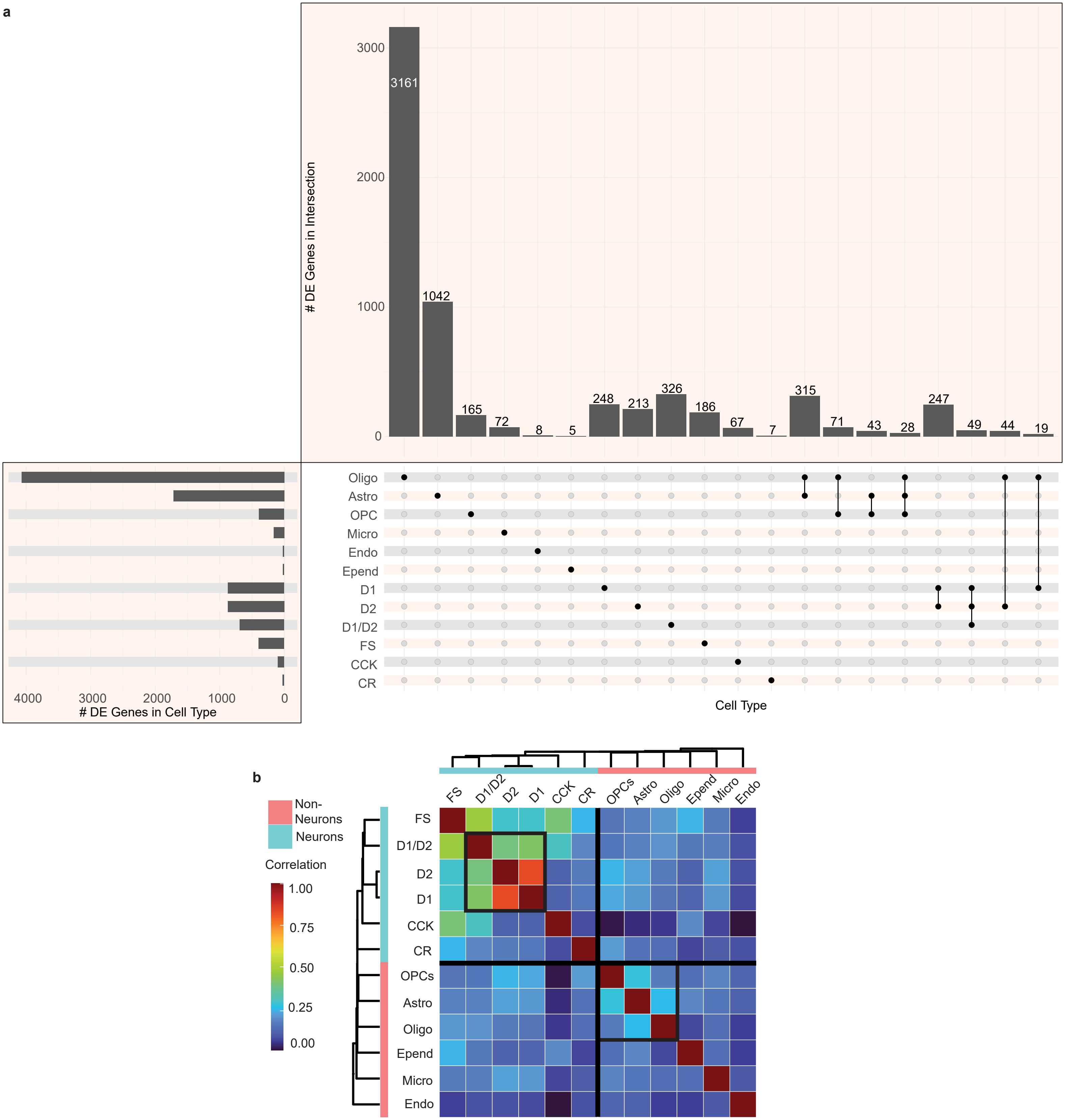

Supplementary Figure 5: **a** Number of genes with expression significantly associated with AUD (FDR < 0.05) in several combinations of cell types. Top bars indicate number of differentially expressed (DE) genes in common (intersection) for the given cell types. Left bars give total number of differentially expressed genes for each cell type. DESeq2 was used for DEG testing. See Supplementary Data 7 for number of individuals tested for each cell type. **b** Heatmap of Pearson correlation of AUD-associated gene expression differences (log2 fold changes) between cell types, hierarchically clustered by Pearson correlation. Color scale indicates Pearson correlation. Red and blue categorization denotes neuronal and non-neuronal cell types. Black-outlined squares indicate groups of cell types with moderate correlation, namely, D1, D2, and D1/D2 neurons, and OPCs, astrocytes, and oligodendrocytes.
Cell type abbreviations: Oligo: Oligodendrocytes, Astro: Astrocytes, OPCs: Oligodendrocyte progenitor cells, Micro: Microglia, Endo: Endothelial Cells, Epend: Ependymal cells, D1: D1 Medium spiny neurons, D2: D2 Medium spiny neurons, D1/D2: D1/D2 hybrid medium spiny neurons, FS: Fast Spiking (PV+) interneurons, LTS: Low-threshold spiking (SST+) interneurons, CCK: CCK+ interneurons, CR: CR+ interneurons
Source data are provided as a Source Data file.

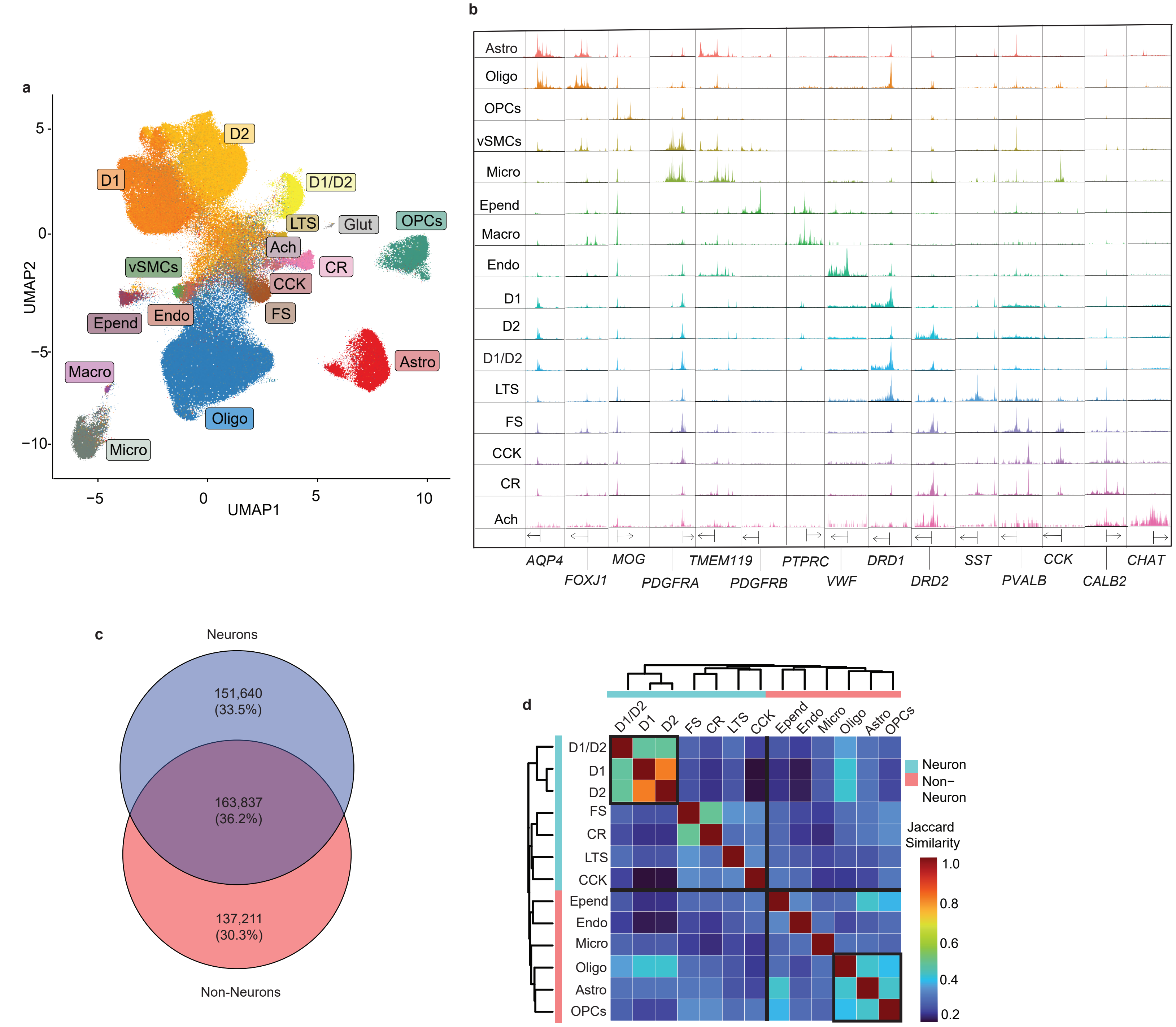

Supplementary Figure 6: **a,** snATAC-seq cell landscape, N = 253,972 cells. UMAP plot displays each cell for which snATAC-seq data was available, clustered based on common set of snATAC-seq peaks (see 'ATAC-seq Integration, Visualization' in Methods). Cell type labels for each cell were provided based on the cell's snRNA-seq data (see Fig. 1). **b** Pseudobulk accessibility profiles for each cell type at canonical marker genes. 5 kilobases on each side of the transcription start site of each gene are shown. **c** Overlap between the union set of open chromatin regions (peaks) from all neuronal cell types (neurons) and the union of peaks from all non-neuronal cell types (non-neurons). The number in the overlapping circle denotes the number of neuronal peaks that overlap in the genome with a non-neuronal peak. The numbers outside the overlap denote the number of peaks in each set that do not overlap with a peak in the other. **d** Heatmap of Jaccard similarity between open chromatin regions in each cell type, hierarchically clustered by Jaccard similarity. Color scale indicates Pearson correlation. Blue and red categorization denotes neuronal and non-neuronal cell types. Black-outlined squares indicate groups of cell types with moderate correlation, namely, D1, D2, and D1/D2 neurons, and OPCs, astrocytes, and oligodendrocytes.

Cell Type Abbreviations:

- Astro: Astrocytes
- CCK: CCK+ interneurons
- CR: CR+ interneurons
- Ach: Cholinergic neurons
- D1: D1 Medium spiny neurons
- D2: D2 Medium spiny neurons
- D1/D2: D1/D2 hybrid medium spiny neurons
- Epend: Ependymal cells
- FS: Fast Spiking (PV+) interneurons
- LTS: Low-threshold spiking (SST+) interneurons
- Micro: Microglia
- vSMCs: Vascular smooth muscle cells
- OPCs: Oligodendrocyte progenitor cells
- Oligo: Oligodendrocytes
- Macro: Macrophages
- Endo: Endothelial Cells

Source data are provided as a Source Data file.

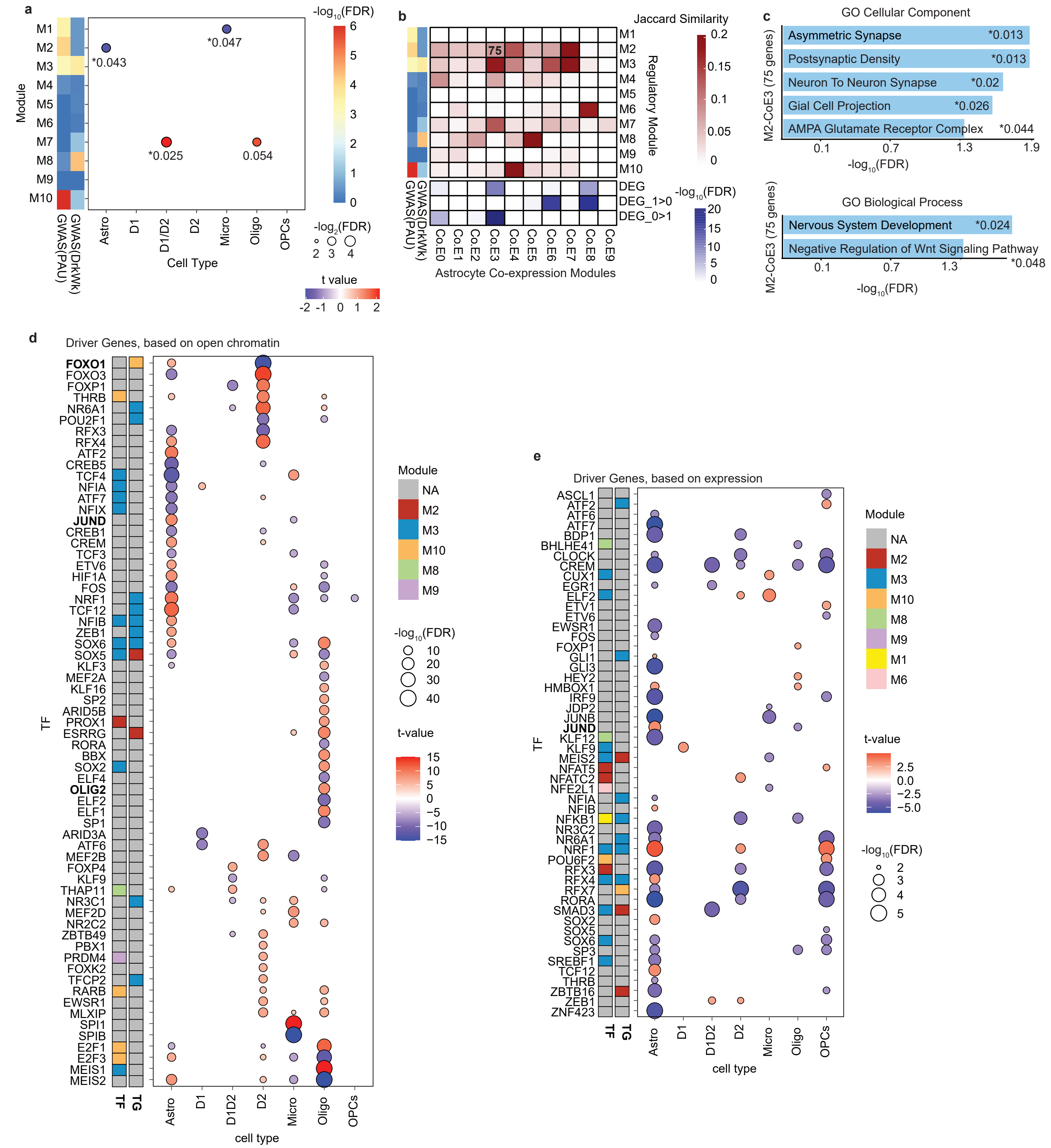

Supplementary Figure 7. **a** Gene regulatory network analysis of samples from individuals with and without AUD comparing the average expression of genes within 10 regulatory modules (M1-M10). Dot size represents Benjamini-Hochberg adjusted FDR and color represents t-value (2-sided t-test) of the difference in average gene expression. Asterisk indicates significance (FDR < 0.05). Left two columns display enrichment of module genes in the set of genes significantly associated with problematic alcohol use (GWAS(PAU)) and drinks per week (GWAS(Drkwk)), using a gene set enrichment test. **b** Astrocyte co-expression modules. Left two columns are the same as in a. Top (red heatmap), Jaccard similarity between genes belonging to the 10 regulatory modules, and 10 co-expression modules (Co.E0-Co.E9), as calculated by WGCNA. Bottom (blue heatmap), Benjamini-Hochberg adjusted FDR of a two-sided t test of the difference in average expression of genes in each co-expression module, between individuals with and without AUD. DEG_1>0 indicates higher expression in samples from individuals with AUD, and DEG_0>1 indicates higher expression in those without AUD. Bolded number "75" indicates number of genes overlapping between the regulatory module and co-expression module. **c** Gene Ontology (GO) functional enrichment for the 75 genes overlapping regulatory module 2 and co-expression module 3 shown in b. Numbers marked by asterisk indicates Benjamini-Hochberg adjusted FDR of enrichment. **d** Dot plot of all driver genes found, using LINGER's driver score, based on chromatin accessibility of target genes of that transcription factor. Size of dot corresponds to FDR and color indicates t-value, from a two-sided t test, of change in driver score between AUD and non AUD individuals. Left two columns correspond to membership in regulatory modules. Genes ordered by cell type with most significant difference in driver score. Bolded genes denote genes highlighted in "Cell Type-specific Gene-Regulatory Mechanisms in AUD" section. **e** Dot plot of all driver genes found, using LINGER's driver score, based on gene expression of target genes of that transcription factor. Size of dot corresponds to FDR and color indicates t-value, as in d, of change in driver score between AUD and control individuals. Left two columns correspond to membership in regulatory modules. Astro: astrocytes; D1: D1 medium spiny neurons; D2: D2 medium spiny neurons; D1D2: D1/D2 hybrid medium spiny neurons; Micro: microglia; Oligo: oligodendrocytes; OPCs: oligodendrocyte progenitor cells. Source data are provided as a Source Data file.

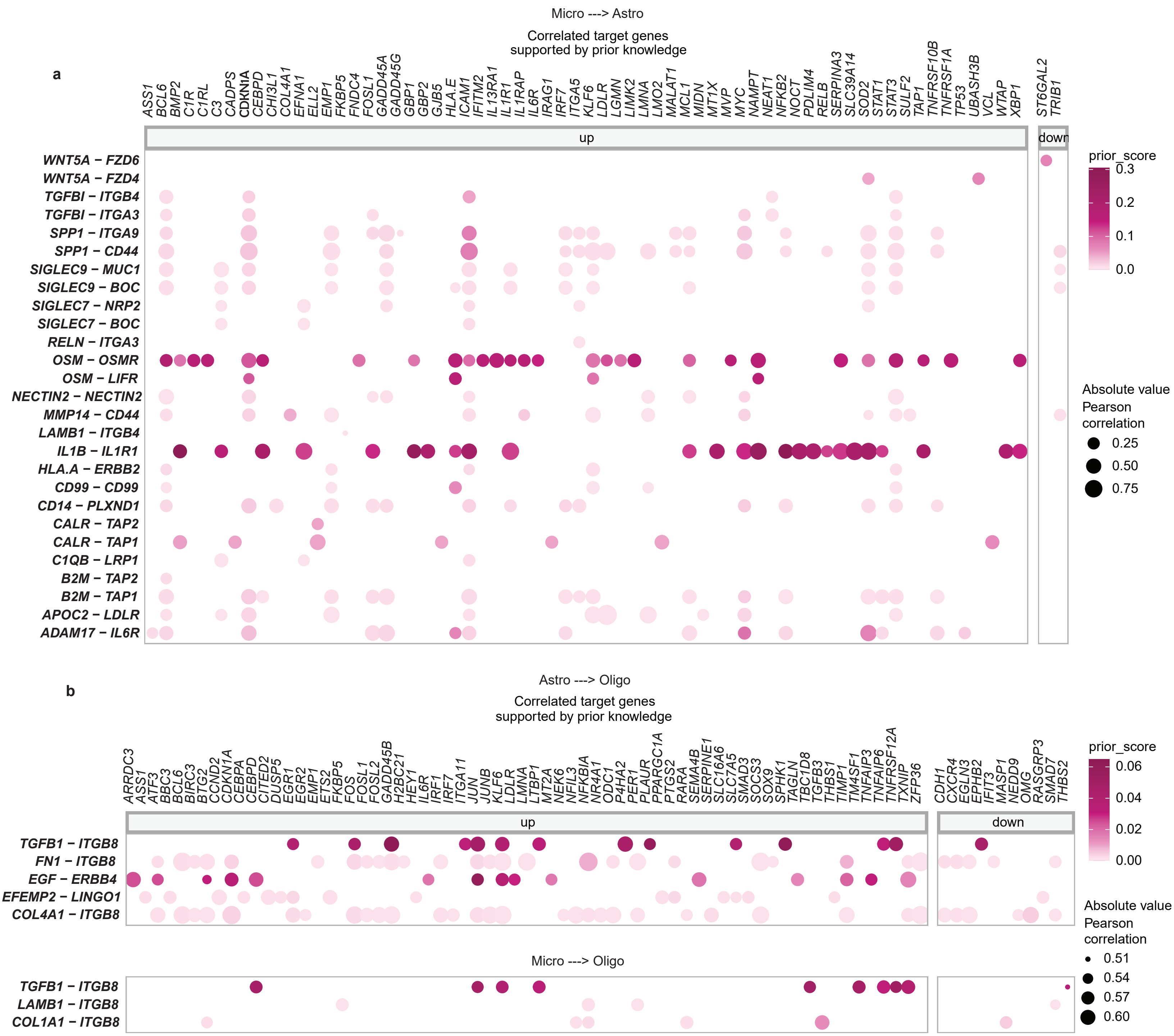

Supplementary Figure 8: Downstream genes of AUD-associated ligand-receptor pairs. **a** Microglia (micro) to astrocytes (astro) ligand-receptor pairs from MultiNicheNet cell-cell communication. All ligand-receptor pairs and target genes with high expression correlation (Spearman or Pearson > 0.50), having some prior knowledge to support their link (in the top 250 predicted target genes for the ligand based on "prior_score" as defined by the MultiNicheNet v2 networks), and being within the top 50 ligand-receptor pairs associated with AUD (as calculated by MultiNicheNet) are shown. Size of dots indicate Pearson correlation between expression of ligand-receptor pair and target gene. Color of dot indicates prior score for link between ligand-receptor and downstream gene. **b** As in a, with astrocytes to oligodendrocytes (oligo) pairs (above) and microglia to oligodendrocyte pairs (below). See Methods and MultiNicheNet documentation for details on prior knowledge database and ligand-receptor pair scoring criteria. Source data are provided as a Source Data file.

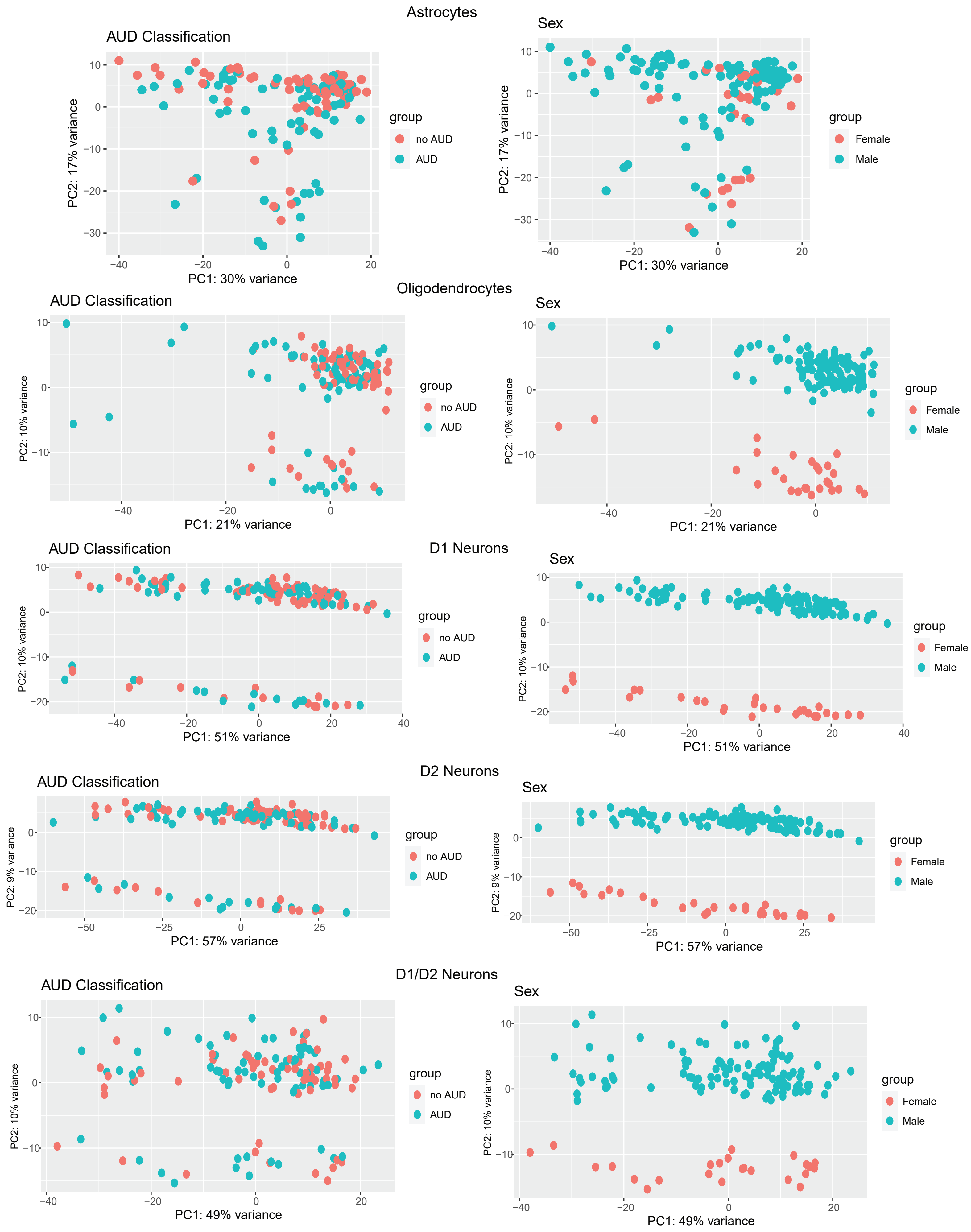

Supplementary Figure 9: Individual samples plotted by top two principal components of pseudobulk-level RNA expression for astrocytes (n=143 individuals), oligodendrocytes (n=143), D1 (n=143), D2, and D1/D2 neurons (n=133), separated by AUD classification and sex. Source data are provided as a Source Data file.

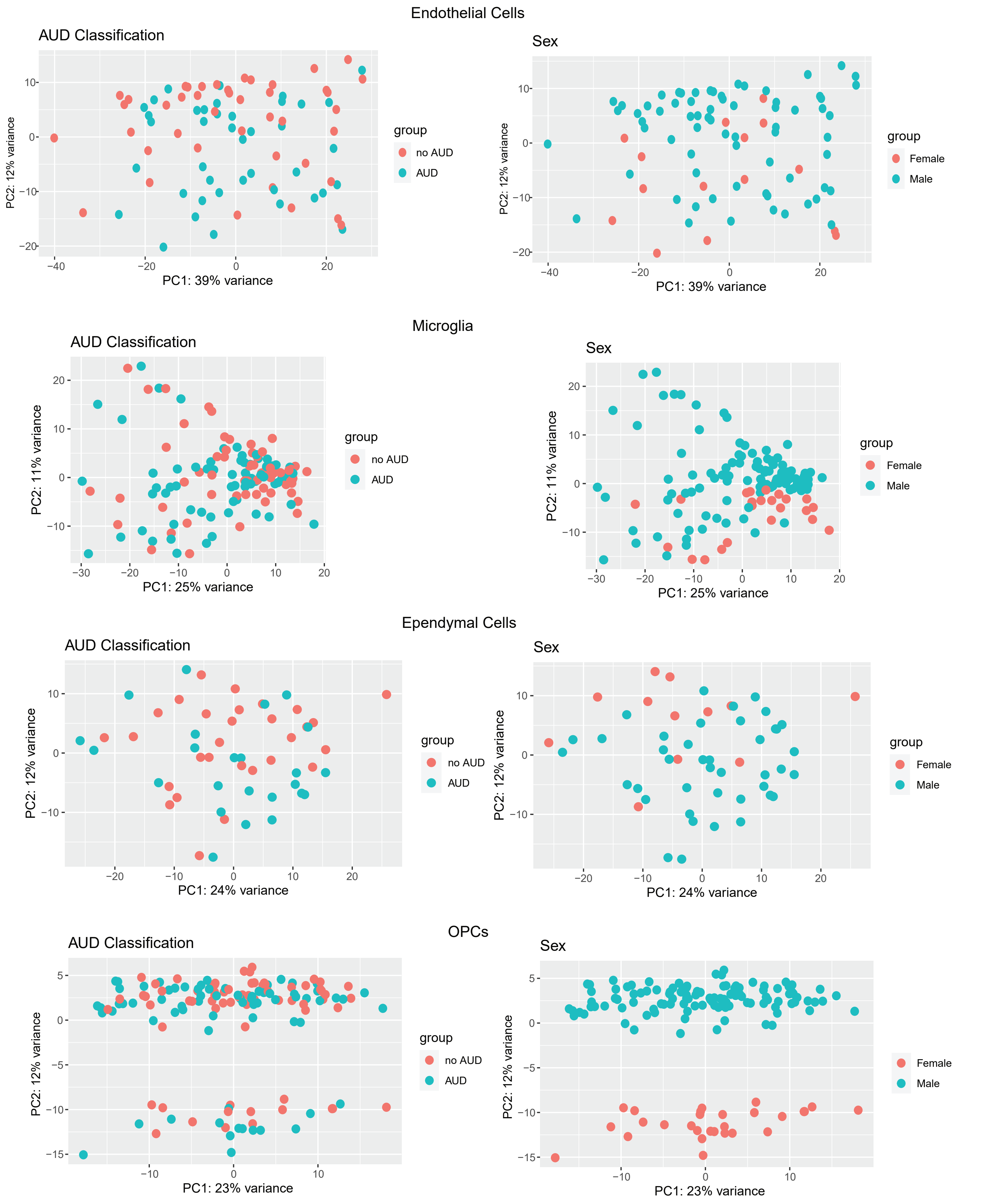

Supplementary Figure 10: Individual samples plotted by top two principal components of pseudobulk-level RNA expression for endothelial cells (n=84 individuals), microglia (n=131), ependymal cells (n=53), and OPCs (n=143), separated by AUD classification and sex. Source data are provided as a Source Data file.

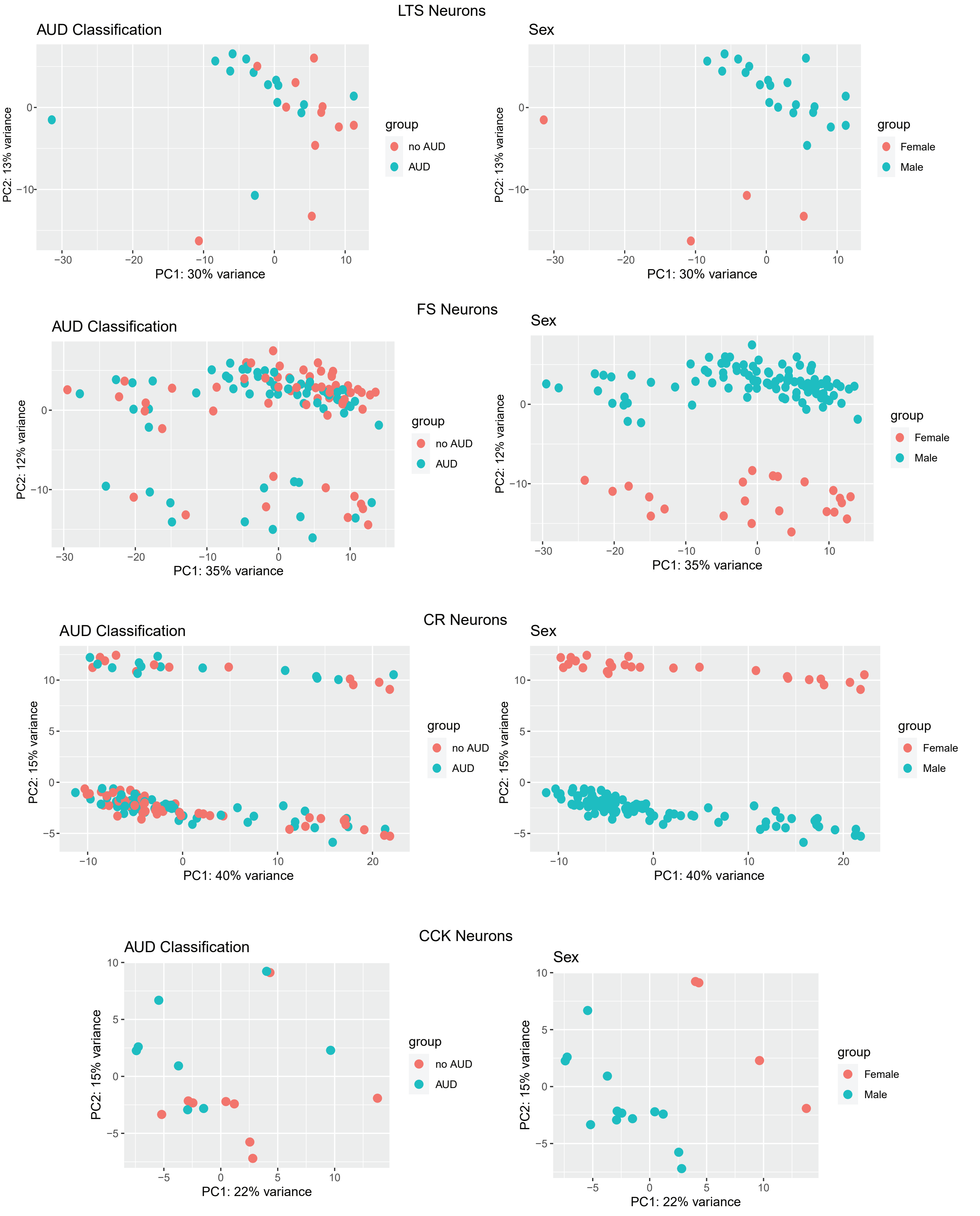

Supplementary Figure 11: Individual samples plotted by top two principal components of pseudobulk-level RNA expression for LTS (n=25 individuals), FS (n=128), CR (n=138), and CCK interneurons (n=17), separated by AUD classification and sex. Source data are provided as a Source Data file.

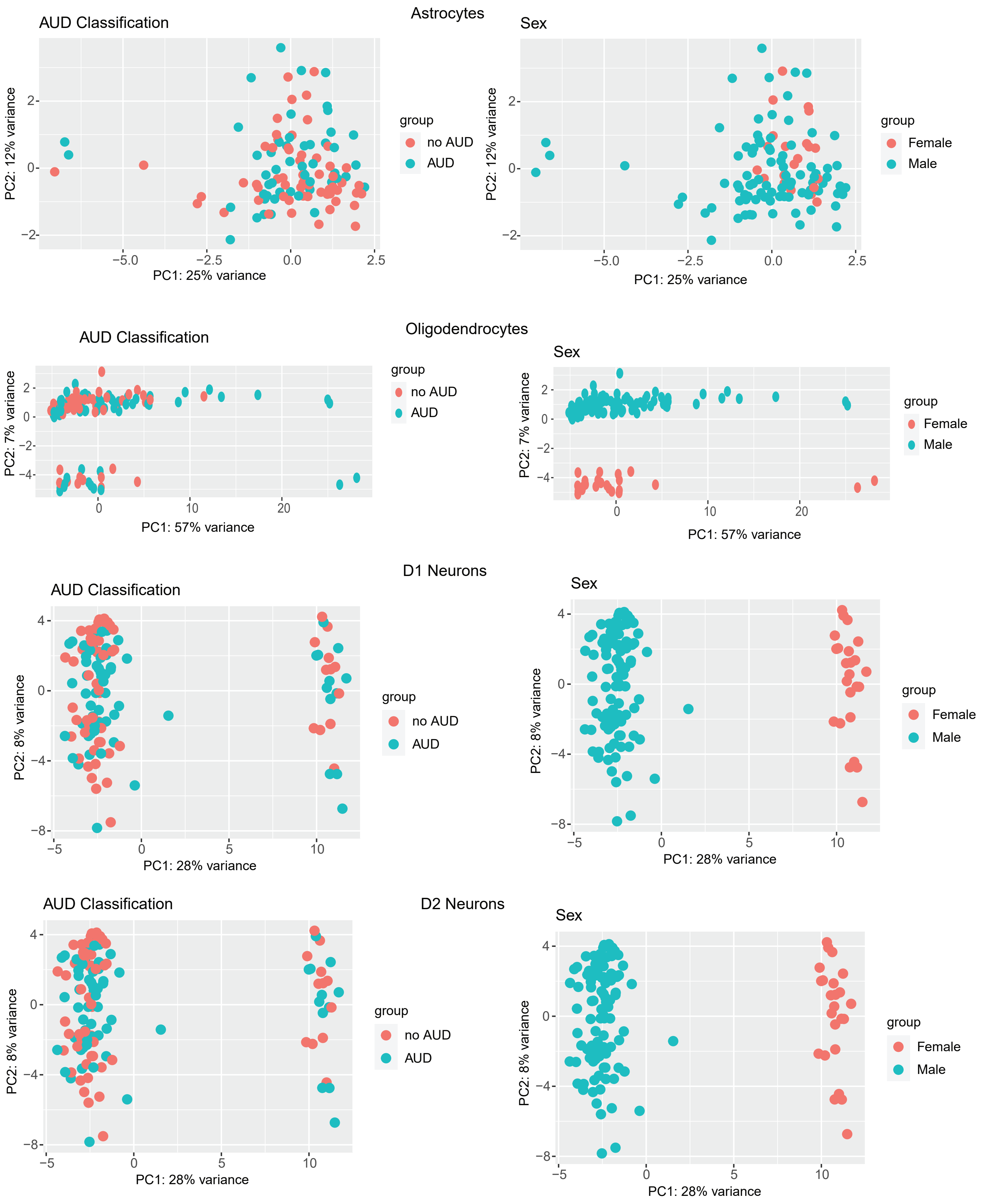

Supplementary Figure 12: Individual samples plotted by top two principal components of pseudobulk-level ATAC counts for astrocytes (n=110 individuals), oligodendrocytes (n=130), D1 (n=125), and D2 neurons (n=132), separated by AUD classification and sex. Source data are provided as a Source Data file.

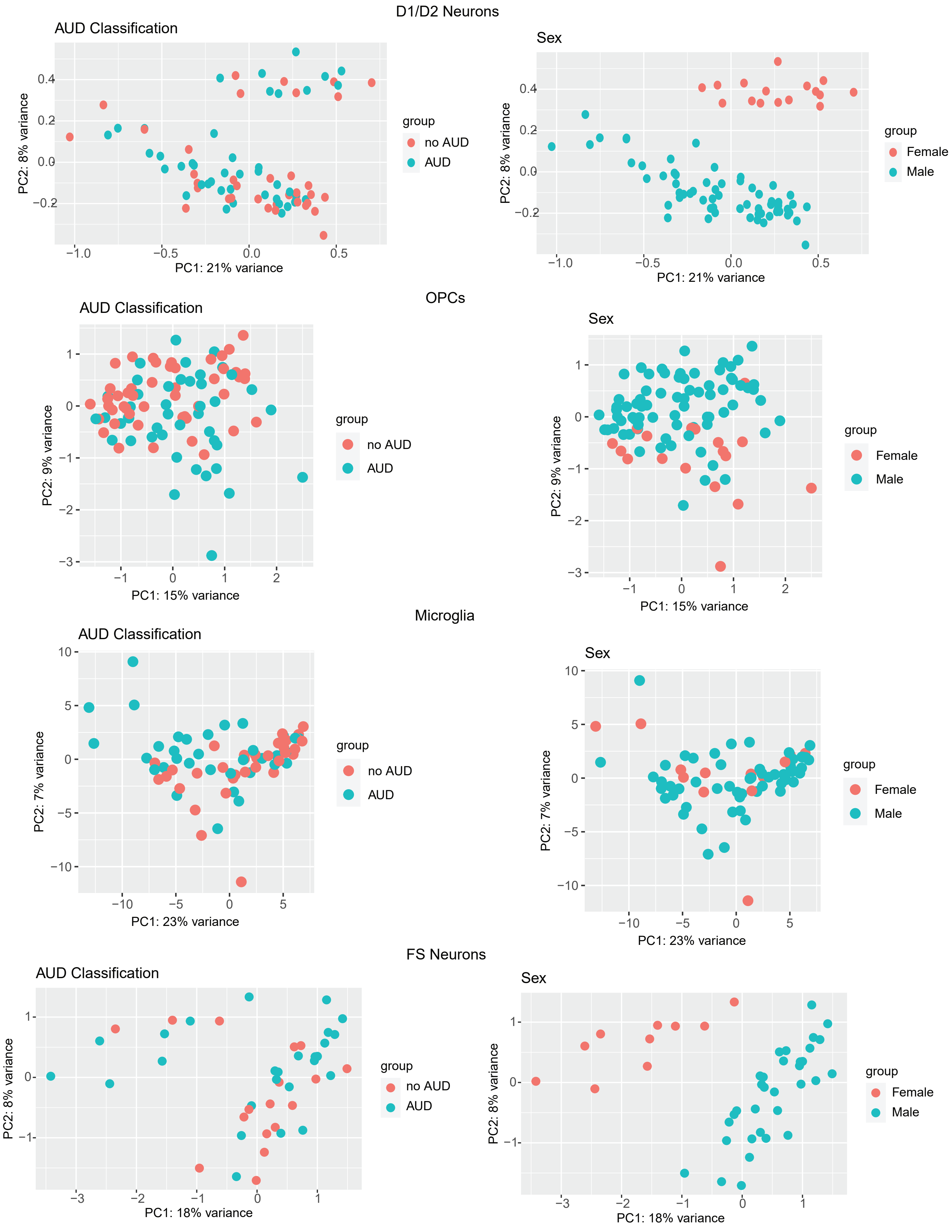

Supplementary Figure 13: Individual samples plotted by top two principal components of pseudobulk-level ATAC counts for D1/D2 neurons (n=79 individuals), OPCs (n=93), microglia (n=70), and FS interneurons (n=43), separated by AUD classification and sex. Source data are provided as a Source Data file.
