# Supplementary material for: Integrated single-cell multiomic profiling of caudate nucleus suggests key mechanisms in alcohol use disorder
Source: Nat Commun. 2025 Oct 13;16:9070. doi: 10.1038/s41467-025-64136-0 (PMC12518533; doi:10.1038/s41467-025-64136-0)
Supplement: Supplementary file 2 — Description of Additional Supplementary Files [file 41467_2025_64136_MOESM2_ESM.pdf]

Description of Additional Supplementary Files

Supplementary Data 1: Sample-level demographics and cell-level information from snRNA-seq results.

Supplementary Data 2: Sample-level demographics and cell-level information from snATAC-seq results.

Supplementary Data 3: Microglia subcluster-specific gene markers, using ROC test.

Supplementary Data 4: Enriched GO pathways in microglia subcluster-specific genes.

Supplementary Data 5: Astrocyte subcluster-specific gene markers, using ROC test.

Supplementary Data 6: Enriched GO pathways in astrocyte subcluster-specific genes.

Supplementary Data 7: Number of differentially expressed genes for each cell type.

Supplementary Data 8: Correlation between cell types of log2 fold changes of genes between AUD and non-AUD individuals, from differential expression analysis.

Supplementary Data 9: Gene set enrichment analysis results for each cell type, using genes ranked by differential expression log2 fold change.

Supplementary Data 10: Jaccard Index between cell types of open chromatin regions.

Supplementary Data 11: Correlation between cell types of RNA and chromatin accessibility counts for all genes.

Supplementary Data 12: Number of differentially accessible regions for each cell type.

Supplementary Data 13: Enrichment of differentially expressed genes with alcohol-related GWAS genes.

Supplementary Data 14: TF and TG Regulatory Modules Assignments.

Supplementary Data 15: Co-expression modules in astrocytes.

Supplementary Data 16: Differential chromVar motif activity in astrocytes.

Supplementary Data 17: Differential chromVar motif activity in oligodendrocytes.

Supplementary Data 18: Microglia-astrocyte-oligodendrocyte cell-cell communication results (MultiNicheNet).

Supplementary Data 19: Barcode-level quality control metrics used for HT assay snRNA-seq data, for each sequencing pool.

Supplementary Data 20: Barcode-level quality control metrics used for multiome assay snRNA-seq + snATAC-seq data, for each sequencing pool.
