# Supplementary material for: Integrated single-cell multiomic profiling of caudate nucleus suggests key mechanisms in alcohol use disorder
Source: Nat Commun. 2025 Oct 13;16:9070. doi: 10.1038/s41467-025-64136-0 (PMC12518533; doi:10.1038/s41467-025-64136-0)
Supplement: Supplementary file 4 — Reporting Summary [file 41467_2025_64136_MOESM4_ESM.pdf]

nature portfolio

Corresponding author(s): Yunlong Liu, Howard J. Edenberg

Last updated by author(s): Sep 3, 2025

Reporting Summary

Nature Portfolio wishes to improve the reproducibility of the work that we publish. This form provides structure for consistency and transparency in reporting. For further information on Nature Portfolio policies, see our Editorial Policies and the Editorial Policy Checklist.

Statistics

For all statistical analyses, confirm that the following items are present in the figure legend, table legend, main text, or Methods section.

n/a	Confirmed	
☐	☒	The exact sample size (*n*) for each experimental group/condition, given as a discrete number and unit of measurement
☐	☒	A statement on whether measurements were taken from distinct samples or whether the same sample was measured repeatedly
☐	☒	The statistical test(s) used AND whether they are one- or two-sided<br>*Only common tests should be described solely by name; describe more complex techniques in the Methods section.*
☐	☒	A description of all covariates tested
☐	☒	A description of any assumptions or corrections, such as tests of normality and adjustment for multiple comparisons
☐	☒	A full description of the statistical parameters including central tendency (e.g. means) or other basic estimates (e.g. regression coefficient) AND variation (e.g. standard deviation) or associated estimates of uncertainty (e.g. confidence intervals)
☐	☒	For null hypothesis testing, the test statistic (e.g. *F*, *t*, *r*) with confidence intervals, effect sizes, degrees of freedom and *P* value noted<br>*Give P values as exact values whenever suitable.*
☒	☐	For Bayesian analysis, information on the choice of priors and Markov chain Monte Carlo settings
☒	☐	For hierarchical and complex designs, identification of the appropriate level for tests and full reporting of outcomes
☐	☒	Estimates of effect sizes (e.g. Cohen's *d*, Pearson's *r*), indicating how they were calculated

Our web collection on statistics for biologists contains articles on many of the points above.

Software and code

Policy information about availability of computer code

Data collection	GEM generation and barcoding was performed by the 10x Genomics Chromium X Controller. All sequencing libraries were examined on the Agilent Bioanalyzer 2000 and sequenced on the Illumina NovaSeq 6000.

Data analysis	Genotype data processing and imputation: Eagle v2 was used to phase the genotypes and Minimac4 v1.2.4 was used for imputation.<br>Single-nuclei multiome raw data processing: Cell Ranger Count (cellranger-7.0.1)<br>Single-nuclei multiome raw data processing: Cell Ranger ARC (cellranger-arc-2.0.0)<br><br>The following are command line tools used for the following:<br>Demultiplexing: demulxlet v1.0-5<br>bam file processing: sinto v0.10.1, samtools v1.17<br>Variant calling: Sentieon germline variant calling pipeline<br><br>The following are R (version 4.2.1) packages used for analysis:<br>Single-nuclei RNA-seq analysis: Seurat v5.0.0<br>Single-nuclei ATAC-seq analysis: Signac v1.12.0<br>Differential expression/accessiblity analyses: DESeq2 v1.42.1<br>Gene set enrichment analysis: fgsea 1.26.0<br>Motif enrichment: chromVAR v1.22.1<br>Cell-cell communication: MultiNicheNet v1.0.3<br><br>Gene regulatory network inference: LINGER v1.0.0 (see Yuan & Duren, 2024)

For manuscripts utilizing custom algorithms or software that are central to the research but not yet described in published literature, software must be made available to editors and reviewers. We strongly encourage code deposition in a community repository (e.g. GitHub). See the Nature Portfolio guidelines for submitting code & software for further information.

Data

Policy information about availability of data

All manuscripts must include a data availability statement. This statement should provide the following information, where applicable:

- Accession codes, unique identifiers, or web links for publicly available datasets
- A description of any restrictions on data availability
- For clinical datasets or third party data, please ensure that the statement adheres to our policy

The GWAS datasets utilized in this study were obtained from:
1) Saunders, et al., summary statistics of which can be found at the Data Repository for the University of Minnesota [https://doi.org/10.13020/przg-dp88]
2) Zhou, et al., of which full summary-level information can be found at Yale School of Medicine [https://medicine.yale.edu/lab/gelernter/stats/] and dbGaP (accession number phs001672 [https://www.ncbi.nlm.nih.gov/projects/gap/cgi-bin/study.cgi?study_id=phs001672]).

The data generated here, including raw sequencing data in the form of BAM files and processed data in the form of Seurat RDS objects are accessible through GEO series Accession Number GSE277313 [https://www.ncbi.nlm.nih.gov/geo/query/acc.cgi?acc=GSE277313]
All other data that support the findings of this study are either listed in Supplementary Data or as Source Data. Code used to carry out the analyses is available at https://github.com/nick-c-green/Caudate-scMultiome.

Research involving human participants, their data, or biological material

Policy information about studies with human participants or human data. See also policy information about sex, gender (identity/presentation), and sexual orientation and race, ethnicity and racism.

Reporting on sex and gender	Sex was used as a covariate in several analyses (see Online Methods). Sex was determined based on self-reporting. Of the 170 individuals sequenced in both HT and multiome assays, 131 were reported as males and 39 as female. Although sex was used as a covariate, sex-specific analyses were not performed due to the much smaller number of females in the study, as compared to males.
Reporting on race, ethnicity, or other socially relevant groupings	"Ethnicity"/"Ethnic origin" was determined by self-report. Of the 170 individuals sequenced in both HT and multiome assays, 169 were reported as European origin, and 1 as Asian. Although ethnic origin was used as a covariate in several analyses (including differential expression/accessibility, cell-type/subtype proportion, cell-cell communication, eQTL, motif enrichment, and gene regulatory network analysis), ethnicity-specific analyses were not performed due to the predominantly European dataset.
Population characteristics	Covariate-relevant population characteristics: Adult males or females aged 18+, either medically healthy or having a diagnosis of alcohol use disorder.
Recruitment	Brain donors were recruited by the New South Wales Brain Resource Tissue Centre (NSWBTRC) from the University of Sidney. The NSWBTRC operates in collaboration with the National Institute of Alcohol Abuse and Alcoholism in the US and has ethics approval from the University of Sydney. Specifically, the donor recruitment program (called the 'Using our Brains' donor program) was established in 2002 as a pre-mortem consent program inviting members of the community (those living within the NSW Sydney Metropolitan, Hunter or Illawarra Region) to donate their brain to neuroscience research after they die. Their collection focuses on controls, alcohol-related brain damage and mental illness, specifically schizophrenia. The NSWBTRC encourages those affected with specific brain disorders and those who are medically healthy to donate. Those eligible to be a donor: 1) are aged 18 years and over, 2) live in NSW Sydney Metropolitan, Hunter or Illawarra Region, 3) are not a Whole Body Donor (different to organ donation), 4) do not have an infectious disease such as Hepatitis, HIV, AIDS, CJD, 5) have not been diagnosed with a brain tumour, stroke, epilepsy, or other neurological illness, 6) do not suffer from a serious head injury with loss of consciousness. Eligible donors complete a pre-screen via telephone or the online enquiry form, complete a 'consent kit' that contains detailed information on the Using our Brains program and consent forms.

	Consented donors are contacted annually to update their contact, medical and lifestyle details.
Ethics oversight	The NSWBTRC has ethics approval from the University of Sydney.

Note that full information on the approval of the study protocol must also be provided in the manuscript.

Field-specific reporting

Please select the one below that is the best fit for your research. If you are not sure, read the appropriate sections before making your selection.

☒ Life sciences ☐ Behavioural & social sciences ☐ Ecological, evolutionary & environmental sciences

For a reference copy of the document with all sections, see nature.com/documents/nr-reporting-summary-flat.pdf

Life sciences study design

All studies must disclose on these points even when the disclosure is negative.

Sample size	Sample sizes were not predetermined based on statistical methods, but were chosen according to the standards of the field (at least three independent biological replicates for each condition, here, 74 samples with AUD and 69 controls) which generated a sufficient number of replicates and gave sufficient statistics for the effect sizes of interest.
Data exclusions	170 postmortem brains were originally sequenced in both HT and multiome assays. After demultiplexing, seven donors with less than 200 cell barcodes assigned were removed from all further analyses; all of the remaining 163 donors had over 1,000 barcodes. Low-quality barcodes were removed based on criteria detailed in Online Methods. Following clustering and cell type identification, 20 samples with a proportion of glutamatergic neurons greater than 10% were removed, because such a cell-type composition indicates potential contamination with non-caudate tissue, leaving 143 samples for the remaining downstream analyses. Samples were removed on a cell type-specific basis for the differential expression/accessibility analyses if the sample contained less than 50 cells of that cell type. See Methods for more details.
Replication	To ensure reproducibility, a robust sample size was utilized (see above). Two technical replicates were successfully sequenced for each pool (see Methods). The donors in each pool were both condition (control or AUD) and sex balanced. At each step, the quality of cDNA, ATAC library and cDNA library was examined by Bioanalyzer 2000. To ensure robust and reproducible results, low-quality samples were removed at relevant steps of analysis (see above).
Randomization	Samples were assigned to either case or control groups based on prior alcohol use disorder diagnosis. Subjects in each assay pool were both case/control and sex balanced. Sex, age, and ethnic origin were controlled for in cell type/subtype proportion testing, differential expression, differential accessibility, cell-cell communication, motif enrichment, and gene regulatory network analyses.
Blinding	Identifying information for each patient was hidden from all those performing analyses. Only a numeric ID and information on AUD status and relevant covariates were shared with the research team.

Reporting for specific materials, systems and methods

We require information from authors about some types of materials, experimental systems and methods used in many studies. Here, indicate whether each material, system or method listed is relevant to your study. If you are not sure if a list item applies to your research, read the appropriate section before selecting a response.

Materials & experimental systems

n/a	Involved in the study	
☒	☐	Antibodies
☒	☐	Eukaryotic cell lines
☒	☐	Palaeontology and archaeology
☒	☐	Animals and other organisms
☒	☐	Clinical data
☒	☐	Dual use research of concern
☒	☐	Plants

Methods

n/a	Involved in the study	
☒	☐	ChIP-seq
☒	☐	Flow cytometry
☒	☐	MRI-based neuroimaging

Plants

Seed stocks

Report on the source of all seed stocks or other plant material used. If applicable, state the seed stock centre and catalogue number. If plant specimens were collected from the field, describe the collection location, date and sampling procedures.

Novel plant genotypes

Describe the methods by which all novel plant genotypes were produced. This includes those generated by transgenic approaches, gene editing, chemical/radiation-based mutagenesis and hybridization. For transgenic lines, describe the transformation method, the number of independent lines analyzed and the generation upon which experiments were performed. For gene-edited lines, describe the editor used, the endogenous sequence targeted for editing, the targeting guide RNA sequence (if applicable) and how the editor was applied.

Authentication

Describe any authentication procedures for each seed stock used or novel genotype generated. Describe any experiments used to assess the effect of a mutation and, where applicable, how potential secondary effects (e.g. second site T-DNA insertions, mosiacism, off-target gene editing) were examined.
